# Oxidative Damage Does Not Occur in Striped Hamsters Raising Natural and Experimentally Increased Litter Size

**DOI:** 10.1371/journal.pone.0141604

**Published:** 2015-10-27

**Authors:** Xiao-Ya Zhao, Ji-Ying Zhang, Jing Cao, Zhi-Jun Zhao

**Affiliations:** College of Life and Environmental Science, Wenzhou University, Wenzhou 325035, China; Institute of Zoology, CHINA

## Abstract

Life-history theory assumes that animals can balance the allocation of limited energy or resources to the competing demands of growth, reproduction and somatic maintenance, while consequently maximizing their fitness. However, somatic damage caused by oxidative stress in reproductive female animals is species-specific or is tissue dependent. In the present study, several markers of oxidative stress (hydrogen peroxide, H_2_O_2_ and malonadialdehyde, MDA) and antioxidant (catalase, CAT and total antioxidant capacity, T-AOC) were examined in striped hamsters during different stages of reproduction with experimentally manipulated litter size. Energy intake, resting metabolic rate (RMR), and mRNA expression of uncoupling protein 1 (UCP_1_) in brown adipose tissue (BAT) and UCP_3_ in skeletal muscle were also examined. H_2_O_2_ and MDA levels did not change in BAT and liver, although they significantly decreased in skeletal muscle in the lactating hamsters compared to the non-reproductive group. However, H_2_O_2_ levels in the brain were significantly higher in lactating hamsters than non-reproductive controls. Experimentally increasing litter size did not cause oxidative stress in BAT, liver and skeletal muscle, but significantly elevated H_2_O_2_ levels in the brain. CAT activity of liver decreased, but CAT and T-AOC activity of BAT, skeletal muscle and the brain did not change in lactating hamsters compared to non-reproductive controls. Both antioxidants did not change with the experimentally increasing litter size. RMR significantly increased, but BAT UCP_1_ mRNA expression decreased with the experimentally increased litter size, suggesting that it was against simple positive links between metabolic rate, UCP_1_ expression and free radicals levels. It may suggest that the cost of reproduction has negligible effect on oxidative stress or even attenuates oxidative stress in some active tissues in an extensive range of animal species. But the increasing reproductive effort may cause oxidative stress in the brain, indicating that oxidative stress in response to reproduction is tissue dependent. These findings provide partial support for the life-history theory.

## Introduction

In mammals, reproduction, in particularly lactation, is the most energetically demanding period of a female, during which a dramatic increase in food intake occurs [1]. It is well believed that the maximum food intake is limited during peak lactation, and the limitation is of importance because it defines an envelope within which all the competing biological functions are constrained [2]. Life-history theory assumes that animals can balance the allocation of limited energy or resources to the competing demands of growth, reproduction, and somatic maintenance while consequently maximizing their fitness [3]. However, the cost of reproduction may have negative effects on other life-history components, for example the somatic damage caused by oxidative stress [4,5].

Oxidative stress is an extreme process that occurs when there is a serious overproduction of reactive oxygen species (ROS) relative to the protection and repair mechanisms [6–8]. ROS are produced primarily within mitochondria and unavoidable by-products of aerobic metabolism [8,9]. The oxidative stress life-history theory proposes that ROS are produced in direct proportion to metabolic rate as an inevitable consequence of the molecular functioning of mitochondria and the electron transport chain [10]. An animal usually has much higher metabolic rate during lactation, which may cause an increase in ROS production and consequently result in oxidative stress [3,4,7,11–14].

However, it has been previously found that in a variety of animals during lactation or cold exposure the increased metabolic rates do not definitely cause oxidative stress, which is against a simple relationship between metabolic rate and oxidative stress [4,6–8,10,15–21]. Female rodents during lactation do not show a consistent increase in oxidative stress during lactation compared to the non-reproductive females [5,22]. Experimentally increasing reproductive load doesn’t cause oxidative stress in female house mice (*Mus musculus domesticus*) [6,18]. This suggests that the relationship between metabolic rate and the magnitude of oxidative stress is not obvious.

There are two possible reasons for the inconsistency of metabolism and oxidative stress. First, regulations of antioxidants activity may serve to maintain oxidant-antioxidant balance and do not necessarily indicate oxidative stress [4,7]. It has been proposed that the changes leading to an increase in ROS production subsequently upregulate antioxidant defence activity, which consequently decreases oxidative stress [1]. Second, it has been well believed that ROS are produced during oxidative phosphorylation in the electron transport chain in mitochondria, during which a high membrane potential across the mitochondrial inner membrane increases the rate of ROS production [23,24]. Uncoupling proteins (UCPs) decrease the membrane potential, thereby greater uncoupling should lead to lower ROS [24]. This means that higher metabolic rate may have minimal impact on oxidative stress in the animals with more expression of uncoupling proteins [8,10,25–27]. During the last decade, a number of studies have been conducted to test the “oxidative stress life-history theory” in a variety of animal species [3,5,8,10,22,28]. The data from these studies are consistent with either of the two explanations, but it reflects significant species-specific changes in both ROS and antioxidants in response to reproduction [3,28]. There are also notable differences in oxidative stress and antioxidants among the different tissues, including brown adipose tissue (BAT), liver, skeletal muscle, etc [3,5,10,22,28]. It is unclear if this reflects a general pattern across animal species. Thus, the studies in more animal species should be helpful to test the relationship between metabolic rate and the magnitude of oxidative stress.

The striped hamster, *Cricetulus barabensis* (Pallas 1773), is a major rodent in northern China and is also distributed in Russia, Mongolia and Korea [29]. The reproductive period of the wild hamsters comprises of 10 months (ranging from February to November), during which this species has two reproductive peaks in spring and autumn [30–32]. We have previously observed that the energy budgets of lactating hamsters change with the size of litters, and the sustained maximum energy intake is 5.0 × resting metabolic rate (RMR) during peak lactation [32–34]. The aim of the present study was to test the relationships between energy budget, oxidative stress and antioxidant activity in several active tissues, including BAT, liver, skeletal muscle and the brain, in striped hamsters during lactation. First, we examined the relationships in the hamsters on the different stage of reproduction. In the second experiment, oxidative stress and antioxidants, as well as UCP_1_ and UCP_3_ mRNA expression were examined in the hamsters with experimentally regulated litter size. We hypothesized that striped hamsters could balance the allocation of limited energy to the competing demands of lactation and other life-history components. The cost of reproduction might not lead to the somatic damage caused by oxidative stress.

## Methods and Materials

### Animals and ethics statement

Striped hamsters were obtained from the colony at Wenzhou University, which was comprised of the descendants of animals initially trapped in farmland in the center of Hebei Province (115°13’E, 38°12’S), on the North China Plain. Animals were individually housed in plastic cages (29 × 15 × 18 cm) with sawdust bedding in a 12L:12D photoperiod (12:12h light-dark cycle with lights on at 0800h). Room temperature was kept constant at 23 ± 1°C. All animals had *ad libitum* access to a rodent diet (standard rodent chow; Beijing KeAo Feed Co., Beijing, China) and tap water. The field studies involving hamster capture, the protocol and the experimental procedures were approved by the Animal Care and Use Committee (ACUC) of Wenzhou University. The ACUC also granted permission to access the land where hamsters were captured.

**Experiment 1** was designed to examine energy intake, oxidative stress and antioxidant in striped hamsters over the pre-, peak- and post-lactation period. Forty-eight virgin female hamsters were randomly assigned into one of three groups: non-reproductive group (NR, *n* = 8), peak-lactation group (peak-L, *n* = 20) and post-lactation group (post-L, *n* = 20). The females in the peak and post-lactation groups were paired with males for a week, at which point the males were removed. Nine and eight females were pregnant and gave their birth in the peak-L and post-L groups, respectively. The offspring were weaned on day 17 of lactation, and females in the post-L group continued to be housed for two weeks after pups were weaned.

**Experiment 2** was designed to examine the effects of experimentally increasing or decreasing litter size on oxidative stress, antioxidant, RMR and UCP_1_ and UCP_3_ mRNA expression. Females were paired with males according to the same methods that were used in experiment 1. There were no measurements on females and pups following parturition. On day 5 of lactation, females were randomly assigned into one of three groups: a control group, litter size was regulated to 4 for each female (LS = 4, *n* = 8), and decreasing and increasing litter size groups, within which litter size was experimentally deceased to 1 (LS = 1, *n* = 8) and increased to 8 (LS = 8, *n* = 9), respectively. Body mass, litter size, litter mass as well as energy intake were measured on day 13–14 of lactation. RMR was measured on day 16 of lactation. The offspring were weaned on day 17 of lactation.

### Body mass, gross energy intake (GEI), litter size and litter mass

Female body mass and litter mass were measured on day 14 of lactation using a sartorious balance (to 0.1 g). GEI was measured over day 13 and 14 of lactation. It was calculated from food intake measurements taken on day 13 and 14 by subtracting uneaten food and food residues mixed in the bedding material with the initial food provided.

### Markers of oxidative stress

Animals were killed by decapitation at the end of the experiment. Interscapular BAT, liver, leg skeletal muscle and the brain of each animal were removed quickly, and stored in liquid nitrogen. As described previously, tissues were homogenized, and homogenates were centrifuged at 3000 g for 15 min and the resultant supernatant kept for subsequent assays [35]. Protein concentration was determined using the method described by Lowry et al. (1951) [36], using bovine serum albumin (BSA) as standard.

The levels of hydrogen peroxide (H_2_O_2_), indicative of the magnitude of oxidative stress, were measured in BAT, liver, skeletal muscle and the brain. H_2_O_2_ levels were analyzed using a commercial kit (Nanjing Jiancheng Bioengineering Institute) in accordance with the manufacturer’s instructions and guidelines. This kit had been proven effective for the striped hamsters [35]. H_2_O_2_ levels (405nm) were expressed as mmol/g protein.

Malonadialdehyde (MDA) was used as an indicator of lipid peroxidation. MDA is the end product of lipid peroxidation and reacts with thibabituric acid (TBA) to produce a pink colored complex with peak absorbance at 532 nm [37–38]. Levels of tissue MDA were measured in the tissues mentioned above, and were expressed as nmol/mg protein.

### Catalase (CAT) and total antioxidant capacity (T-AOC) activity

Both antioxidant were determined using commercial kits (produced by Nanjing Jiancheng Bioengineering Institute), according to the instructions. The activities of the antioxidant enzymes were all expressed as U/mg protein. 1 unit of CAT activity was defined as the decomposition of 1 μmol H_2_O_2_ per min; 1 unit of T-AOC activity was defined as the increment of 0.01 of absorbance optical density (OD) value per min at 37°C [35].

### Resting metabolic rate (RMR)

RMR was measured as the rate of oxygen consumption using an open respiratory system (TSE, Germany). In brief, Air was pumped at a rate of 1000 ml/min through a cylindrical sealed Perspex chamber, which was put in an incubator to control the chamber temperature within ± 0.5°C. Gases leaving the chamber were directed through a drier before entering into an oxygen analyzer (Siemens, Germany). The data were collected every 10s, and the continuous stable minimum readings over 10 min were taken to calculate RMR. RMR was corrected to standard temperature and air pressure (STP) conditions. All measurements were carried out for 3 h between 1000 and 1700 h to correct for a possible effect of the circadian rhythm.

### Real-time RT-qPCR analysis

Total RNA isolation of BAT, liver, skeletal muscle and the brain tissue was carried out using TRIzol Reagent (TAKARA, Dalian, China). Real-time RT-qPCR analysis was carried out as described by Zhao et al. (2014) [39]. RNA concentration and purity were determined by A260 and A280 OD measurements and A260/A280 ratio determination using a Spectrophotometer. Total RNA was reverse transcribed from 2 μg of total RNA in a final reaction volume of 20 μl, using the random primer oligo (dT)_18_ with AMV Reverse Transcriptase (TAKARA). Two μl cDNA samples were taken as a template for the subsequent PCR reaction using gene-specific primers: UCP_1_, forward, 5’- GGGACCATCACCACCCTGGCAAAAA -3’, reverse, 5’- GGCTTTCTGTTGTGGCTAT -3’; UCP_3_, forward, 5’- ATGGATGCCTACAGAACC -3’, reverse, 5’- GACAATGGCGTTTCTCGT -3’. The final reaction volume of 20 μl contained 10 μl of 2× SYBR Premix EX Tag TM (TAKARA), 0.4 μl of the forward and reverse primers, and 2 μl cDNA template. qPCR was performed using Roche LightCycler480 real-time qPCR system (Forrentrasse CH-6343 Rotkreuz, Switzerland). After performing thermal cycling (according to the manufacturer’s protocol), real-time amplification data were gathered by using LightCycler480 software. Gene expression was normalized to internal controls (housekeeping genes, actin) to determine the fold change in gene expression between test and control samples by ΔΔCT method [39,40].

### Statistics

Data are expressed as mean ± s.e.m., and statistical analyses were conducted in SPSS 13.0 statistics software. In experiment 1, body mass, GEI, H_2_O_2_ and MDA levels and CAT and T-AOC activity in NR, peak-L and post-L groups were examined using one-way ANOVA. In experiment 2, effect of litter size on GEI, litter mass, markers of oxidative stress and antioxidant, and UCP_1_ and UCP_3_ mRNA expression were examined also using one-way ANOVA. RMR was analyzed using one-way ANCOVA with body mass as a covariate. The analysis was followed by Tukey's HSD post-hoc tests if required. *P*-values <0.05 were considered statistically significant.

## Results

### Body mass and energy intake

Body mass did not differ significantly among the NR, peak-L and post-L groups (Table 1). The hamsters in peak-L group showed significantly higher GEI than the other two groups, and the differences between the NR and post-L groups were not significant (Table 1).

**Table 1:** Body mass, energy intake in non-reproductive (NR), peak-lactating (peak-L) and post-lactating (post-L) striped hamsters.

	NR	peak-L	post-L	*F*	*P*
Body mass (g)	29.03±0.71	30.82±1.37	29.81±1.16	0.65	0.53
GEI (kJ/d)	66.03±3.56^b^	259.34±20.34^a^	74.90±8.15^b^	63.06	**
Litter size	-	5.0±0.4	-	-	-
Litter mass (g)	-	52.9±4.7	-	-	-
Mean pup mass (g)	-	10.6±0.9	-	-	-

GEI, gross energy intake; Data are means ± s.e.m.

**, significant difference among the three groups (*P*<0.01). Different letters (a or b) on the same row indicate significant difference (*P*<0.05).

### Hydrogen peroxide (H_2_O_2_) and malonadialdehyde (MDA) levels

H_2_O_2_ levels did not differ among the three groups in BAT and liver, whereas they differed significantly in skeletal muscle and the brain. The females in the peak-L group showed significant lower H_2_O_2_ levels in skeletal muscle, but higher levels in the brain compared with those in the NR and post-L groups (Table 2). MDA levels in liver and skeletal muscle were significantly lower in the peak-L and post-L groups than that in the NR group. No difference was observed in MDA levels among the three groups in BAT and brain (Table 2).

**Table 2:** H_2_O_2_ and MDA levels and CAT and T-AOC activity in non-reproductive (NR), peak-lactating (peak-L) and post-lactating (post-L) Striped hamsters.

	NP	peak-L	post-L	*F*	*P*
H_2_O_2_ (mmol/g)					
BAT	45.51±3.82	46.02±2.57	44.98±3.05	0.03	0.97
Liver	17.8±0.83	16.14±0.85	16.13±0.57	1.53	0.24
SM	15.2±1.88^a^	9.14±0.68^b^	12.91±1.11^ab^	5.89	*
Brain	20.85±1.37^ab^	23.42±1.39^a^	18.66±0.74^b^	3.85	*
MDA (nmol/mg)					
BAT	2.08±0.51	1.39±0.44	3.32±0.95	2.23	0.13
Liver	1.40±0.08	1.07±0.11	1.30±0.5	3.81	*
SM	1.72±0.43^a^	0.38±0.04^b^	0.75±0.05^b^	8.31	**
Brain	2.03±0.23	1.96±0.12	1.77±0.10	0.69	0.51
CAT (U/mg)					
BAT	7.97±0.95	7.79±0.71	7.09±1.23	0.22	0.80
Liver	66.09±3.62^a^	52.11±1.81^b^	72.48±2.46^a^	15.63	**
SM	8.40±2.17	13.35±6.98	14.74±4.66	0.39	0.68
Brain	7.07±1.04	5.96±1.33	7.27±0.71	0.43	0.65
T-AOC (U/mg)					
BAT	0.51±0.08	0.43±0.05	0.38±0.07	0.93	0.41
Liver	0.24±0.08	0.17±0.06	0.20±0.07	2.37	0.12
SM	0.18±0.03	0.26±0.12	0.19±0.03	1.51	0.24
Brain	0.55±0.06	0.53±0.03	0.48±0.05	0.59	0.62

Data are means ± s.e.m.

*, significant difference among the three groups (*P*<0.05)

**, *P*<0.01. Different letters (a or b) on the same row indicate significant difference (*P*<0.05)

### Catalase (CAT) and total antioxidant capacity (T-AOC) activity

CAT activity of liver in the peak-L group was 21.2% and 32.2% lower than that in NR and post-L groups, respectively; and the difference between NR and post-L groups was not significant (Table 2). CAT activity of BAT, skeletal muscle and the brain was not different among the three groups. T-AOC activity did not differ among the three groups in any tissues (Table 2).

### Litter size and litter mass

**Fig 1:**
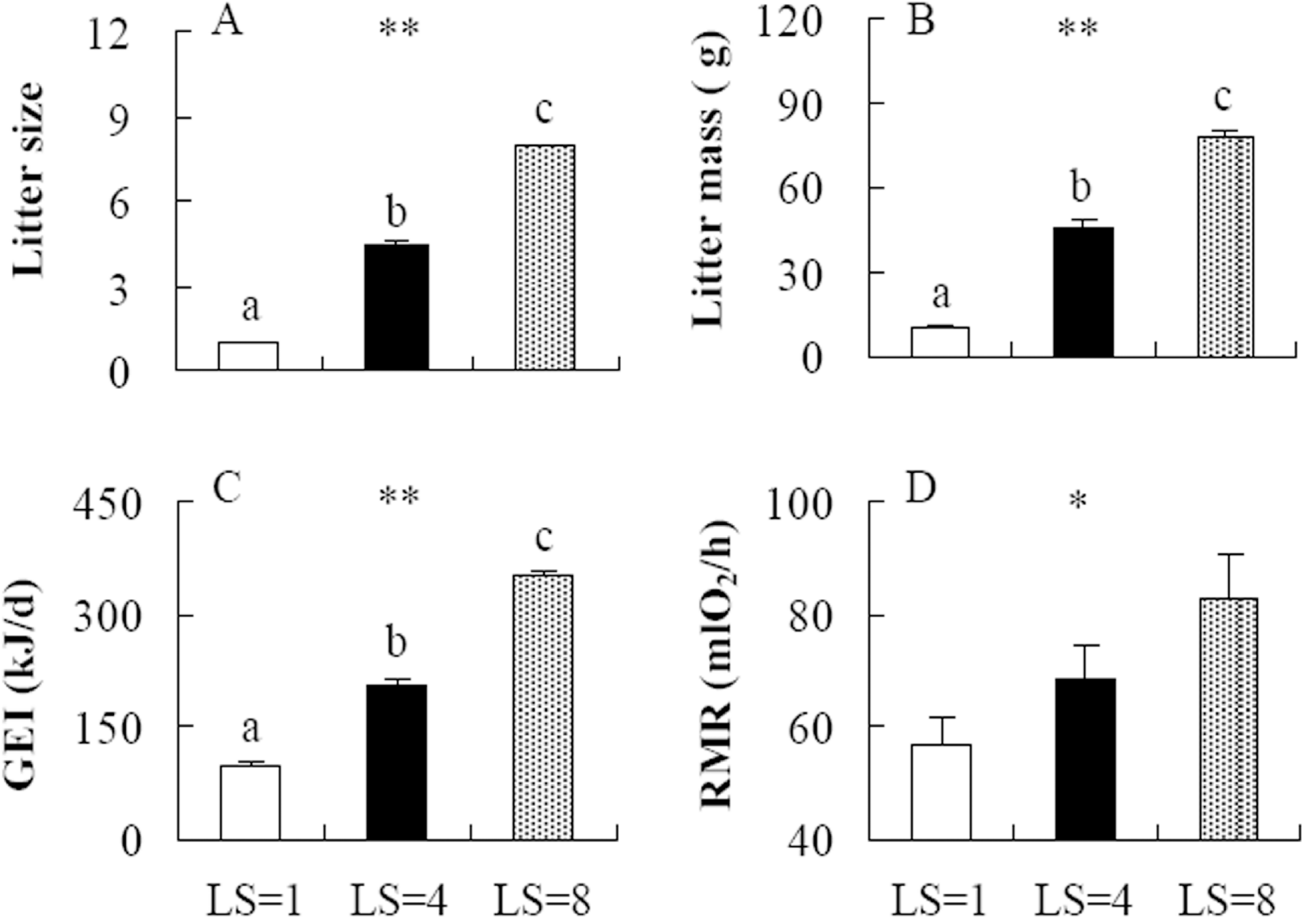
Litter size (A), litter mass (B), gross energy intake (GEI, C) and resting metabolic rate (RMR, D) in striped hamsters during peak lactation. LS = 1, 4 and 8, the females raising 1, 4 and 8 pups throughout lactation. *, significant effect of litter size (*P*<0.05), **, *P*<0.01. Different letters (a, b or c) above the columns indicate significant difference between the groups (*P*<0.05).

Litter size was significantly different among the LS = 1, 4 and 8 groups, and the females raised significantly less pups in the LS = 1 group, but more pups in the LS = 8 group than the females in LS = 4 group (*F*_2,18_ = 900.50, *P*<0.001, post hoc, *P*<0.05, Fig 1A). Consistent with the litter size, litter mass was significantly different among the three groups, and was 76.5% lower in the LS = 1 group but 71.8% heavier in the LS = 8 group compared to that in the LS = 4 group (*F*_2,18_ = 248.53, *P*<0.001, post hoc, *P*<0.05, Fig 1B).

### Gross energy intake (GEI) and resting metabolic rate (RMR)

GEI was significantly affected by litter size, and it decreased by 113.8% in the LS = 1 group, but increased by 72.5% in the LS = 8 group compared with that in the LS = 4 group (*F*_2,18_ = 170.37, *P*<0.001, post hoc, *P*<0.05, Fig 1C). RMR was significantly affected by litter size, by which RMR was 17.49% lower in the LS = 1 group but 21.41% higher in the LS = 8 group compared to that in the LS = 4 group (*F*_2,20_ = 3.99, *P*<0.05, Fig 1D).

### Hydrogen peroxide (H_2_O_2_) levels in the hamsters raising 1, 4 and 8 pups

**Fig 2:**
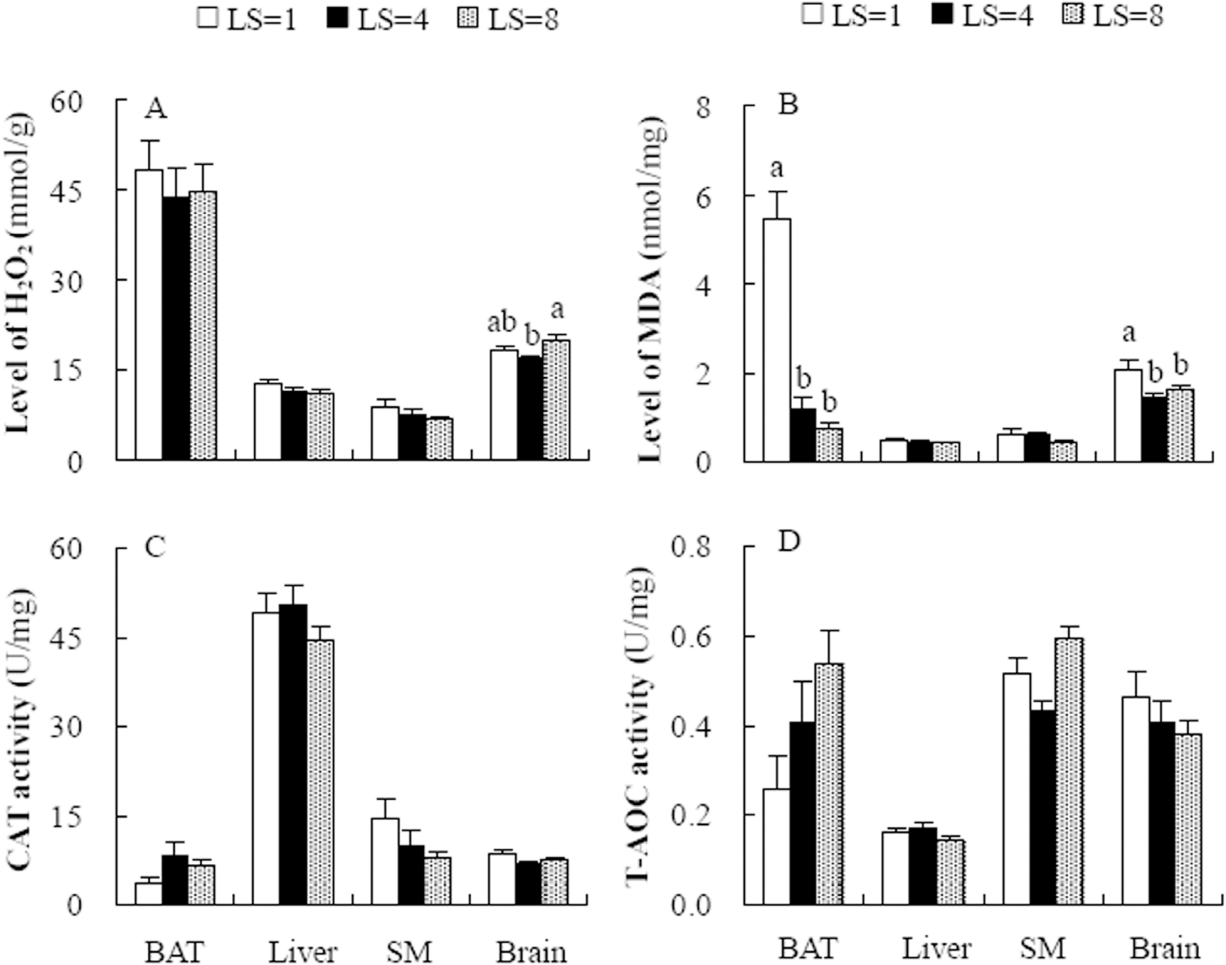
H_2_O_2_ (A) and MDA (B) levels and CAT (C) and T-AOC (D) activity in brown adipose tissue (BAT), liver, skeletal muscle (SM) and the rain in striped hamsters raising 1 (LS = 1), 4 (LS = 4) and 8 (LS = 8) pups. Different letters (a or b) above the columns indicate significant difference between the groups (*P*<0.05).

H_2_O_2_ levels were not different among the LS = 1, LS = 4 and LS = 8 groups in BAT (*F*_2,22_ = 0.27, *P* = 0.77), liver (*F*_2,22_ = 1.83, *P* = 0.18) and skeletal muscle (*F*_2,22_ = 0.07, *P* = 0.39, Fig 2A). In the brain, H_2_O_2_ levels were significantly affected by litter size, and the females in LS = 8 group showed higher H_2_O_2_ levels than those in the LS = 4 group (*F*_2,22_ = 3.61, *P*<0.05, Fig 2A).

### Malonadialdehyde (MDA) levels in the hamsters raising 1, 4 and 8 pups

The three groups did not differ in MDA levels in liver (*F*_2,22_ = 0.52, *P* = 0.61) and skeletal muscle (*F*_2,22_ = 1.67, *P* = 0.34, Fig 2B). Litter size had a significant effect on MDA levels in BAT, by which MDA levels were 78.4% and 86.2% lower in the LS = 4 and LS = 8 groups, respectively, than that in the LS = 1 group (*F*_2,22_ = 45.25, *P*<0.001, post hoc, *P*<0.05, Fig 2B). MDA levels were also significantly affected by litter size in the brain, and the females in LS = 4 and LS = 8 groups showed 28.8% and 21.4% lower MDA levels than those in the LS = 1 group (*F*_2,22_ = 4.19, *P*<0.05, post hoc, *P*<0.05, Fig 2B).

### Catalase (CAT) and total antioxidant capacity (T-AOC) activity in the hamsters raising 1, 4 and 8 pups

Litter size had no significant effect on CAT activity in BAT (*F*_2,22_ = 2.31, *P* = 0.12), liver (*F*_2,22_ = 0.52, *P* = 0.31), skeletal muscle (*F*_2,22_ = 2.02, *P* = 0.16) and the brain (*F*_2,22_ = 2.07, *P* = 0.15, Fig 2C). T-AOC activity was also not affected by litter size in BAT (*F*_2,22_ = 3.03, *P* = 0.07), liver (*F*_2,22_ = 1.81, *P* = 0.19), skeletal muscle (*F*_2,22_ = 2.95, *P* = 0.06) and brain (*F*_2,22_ = 0.88, *P* = 0.43, Fig 2D).

### Uncoupling protein 1 and 3 (UCP_1_ and UCP_3_) mRNA expression

**Fig 3:**
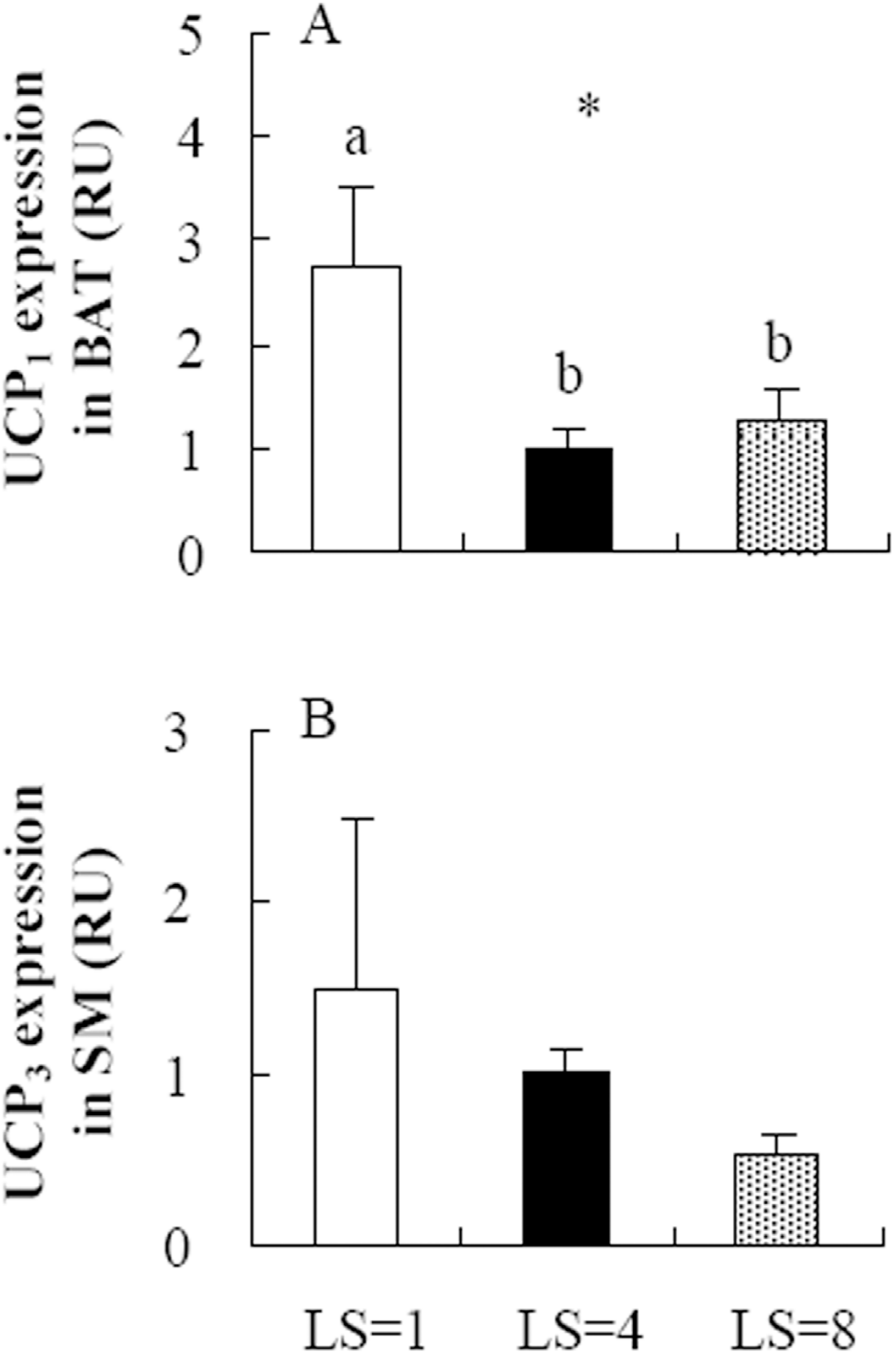
The gene expression of UCP_1_ in brown adipose tissue (BAT, A) and of UCP_3_ in skeletal muscle (SM, B) in striped hamsters raising litters of 1 (LS = 1), 4 (LS = 4) and 8 (LS = 8). *, significant effect of litter size (*P*<0.05). Different letters (a, b) indicate significant difference among the groups (P<0.05).

BAT UCP_1_ mRNA expression was significantly affected by litter size, and it was decreased by 63.6% and 53.5% in the LS = 4 and LS = 8 groups, respectively, compared to that in the LS = 1 group (*F*_2,22_ = 3.73, *P*<0.05, post hoc, *P*<0.05, Fig 3A). No significant effect of litter size on UCP_3_ mRNA expression of skeletal muscle was observed, and there was no difference among the LS = 1, LS = 4 and LS = 8 groups (*F*_2,22_ = 0.73, *P* = 0.49, Fig 3B).

## Discussion

The dramatic increase in energy intake occurs in mammals during lactation to meet the high energy requirements of this most energetically demanding period [1,41]. In the present study, we observed a significant change in the energy intake of striped hamsters during the different stages of reproduction, by which gross energy intake significantly increased in the hamsters during peak lactation compared to that in non-reproductive and post-lactating hamsters. The maximum energy intake during peak lactation was consistent with the studies that were previously performed in the same strain of hamster [32–34]. The females that had experimentally increased litter size elevated energy intake, and weaned larger litters. However, we previously found that the striped hamsters were not able to raise additional pups following parturition [33]. The plausible explanation was that the females might feel stress when we started the manipulation and measurements of litter size and litter mass following the parturition and during the early lactation period, and the mothers cannibalized some of the additional pups, which resulted in reduction of litter size [33]. This might suggest that the captive wild animals were more sensitive to the disturbance of suckling offspring during early lactation than laboratory animals, like mice and rats [42–47]. In his study, we started the manipulation of litter size on day 7 of lactation and the measurements of body mass and litter mass were carried out between day 13 and 15. This might decrease the stress for the females, and consequently the litter size may stay stable throughout the peak lactation.

Life-history theory proposes that animals are able to maximize their fitness by balancing the allocation of limited energy to the competing demands of growth, reproduction, and somatic maintenance [3]. Here, we predicted that the cost of reproduction might not lead to the somatic damage caused by oxidative stress. In this study several markers of oxidative stress (H_2_O_2_ and MDA) in BAT and liver did not change in lactating hamsters compared to non-reproductive and post-lactating groups, indicating that no oxidative stress occurred in these two tissues. This was supported by the studies on lactating house mice [1,4], Mongolian gerbils (*Meriones unguiculatus*) [22], bank voles (*Myodes glaeolus*) [48] and Brandt’s voles (*Lasiopodomys brandtii*) [5]. In skeletal muscle, we even observed that H_2_O_2_ and MDA levels in lactating hamsters decreased by 39.9% and 77.9% compared with non-reproductive controls and by 29.2% and 49.3% relative to post-lactating hamsters. Experimentally increasing litter size did not cause oxidative stress in these tissues. Artificially elevated reproductive effort by adding additional pups decreased ROS levels in some tissues [5,6,10,18]. These findings may suggest that the cost of reproduction has negligible effect on oxidative stress or even attenuated oxidative stress in some inner tissues in extensive animal species.

The brain showed a significant increase in H_2_O_2_ levels in lactating hamsters compared to levels in BAT, liver and skeletal muscle, which were 12.3% and 25.5% higher than non-reproductive and post-lactating groups, respectively (*P*<0.05). Here we can not provide important comparisons across different animal species because to date no one has measured the implication of reproduction on oxidative stress in the brain [10]. Since it has been found that oxidative stress in response to reproductive effort is significantly tissue dependent [5,10,22], the data from this study may imply that the brain may respond to cost of lactation in a different way from other tissues, like BAT, liver and skeletal muscle. Further, levels of H_2_O_2_ and MDA in the brain were affected by litter size, the female hamsters raising 8 pups showed a significant increase in H_2_O_2_ levels compared with those supporting 4 pups. This might suggest that experimentally elevated reproductive effort would result in oxidative stress in the brain in striped hamsters. It indicates that the studies should also pay closer attention to the brain, although a number of studies examined the effect of reproduction on oxidative status in some active tissues, including BAT, liver, skeletal muscle, kidneys [1,3–6,8,10,12,15,16,18,19,21,22,48].

Since ROS are produced continuously during aerobic respiration, organisms utilize a sophisticated set of antioxidants to convert ROS into less active species [9,20,49]. In this study, CAT activity of liver was decreased in lactating striped hamsters compared to that of non-reproductive and post-lactating females, but no difference was observed in T-AOC activity. Both antioxidants were not changed with the experimentally increasing litter size. Inconsistently, activity levels of the antioxidant enzyme (superoxide dismutase, SOD) increased in the liver in house mice with a litter size of eight compared to that of females with two pups or non-reproductive control females [18]. SOD activity also significantly increased in lactating Brant’s voles [5] and Mongolian gerbils [22] compared to that in non-reproductive controls. However, the results from the striped hamsters may be consistent with the findings from other three rodents. This is because in the three studies, oxidative stress in liver was observed to significantly decrease in lactating females relative to non-reproductive groups. In the current study, we observed that oxidative stress of liver did not increase in lactating striped hamsters and those raising larger litter size. In addition, CAT and T-AOC activity in skeletal muscle increased by 58.9% and 44.4% in lactating hamsters in comparison with that of their non-reproductive counterparts. Although the changes were unfortunately not statistically different, it might suggest a potential factor to explain the significant decrease in oxidative stress in this tissue in the lactating hamsters. Similarly, in the brain CAT activity tended to decrease (by 14.4% and 18.0%) in lactating hamsters compared to that in non-reproductive and post-lactating groups, which might partly contribute to the significantly increased oxidative stress in the lactating group. These results may not provide strong evidence for the close relationship between oxidative stress and antioxidants, but they do demonstrate that in an extensive range of species the regulations of antioxidant defense systems occurs to maintain the correct oxidant-antioxidant balance, and consequently protect against oxidative stress [1,4,5,7,18,22].

An animal usually has much higher metabolic rate during lactation. The oxidative stress life-history theory assumes that animals with higher metabolic rate would increase ROS production and consequently result in oxidative stress [3,4,7,10–14]. We have previously found that basal metabolic rate increased by 48% in lactating striped hamsters compared with non-reproductive controls [50]. In this study, RMR decreased by 17.0% in the hamsters raising 2 pups, but increased by 21.4% in the females raising 8 pups in comparison with that of the females supporting 4 pups. This indicated that metabolic rate significantly increased with the experimentally increased litter size. Based on the oxidative stress life-history theory, we predicted that striped hamsters with higher metabolic rate would have more oxidative stress [10]. However, the data from this study was somewhat paradox. This was because in the brain H_2_O_2_ levels were significantly higher in lactating hamsters, and it also increased significantly with increasing litter size, providing a positive support for the theory. But the changes in makers of oxidative stress in skeletal muscle, BAT and liver were not consistent with this theory. It has been proposed that the metabolic rate is not simplistically and positively correlated with ROS production [4–8,10,15–22,51]. The findings of this study not only demonstrated that there is not a strong positive relationship between metabolic rate and ROS levels, but also indicated that the relationship was tissue dependent.

It has been proposed that higher metabolic rate may have minimal impact on oxidative stress in the animals with more expression of uncoupling proteins [8,10,25–27]. This is because those UCPs are responsible for a physiological uncoupling, by which they catalyze basal proton conductance and lower the potential across mitochondria inner membrane, consequently resulting in decrease in ROS production [24,52–55]. UCP_1_ is predominantly expressed in BAT and UCP_3_ is in muscle, and both are involved in basal proton conductance as important mitochondrial inner membrane carrier proteins [23,55,56]. In isolated mitochondria UCP_1_ and UCP_3_ decrease reactive oxygen species production [57,58]. In this study, BAT UCP_1_ mRNA expression was significantly lower in the striped hamsters raising 4 and 8 pups than those supporting 1 pup. UCP_3_ mRNA expression decreased by 31.3% and 63.9% in the hamsters raising 4 and 8 pups, respectively, than that in the hamsters raising 1 pup. UCP_1_ expression in BAT and UCP_3_ expression in skeletal muscle were down-regulated during lactation in other rodents [59,60]. Based on the role of UCP_1_ and UCP_3_ in lowering ROS production, the down-regulation might predict that ROS levels would increase in lactating females. However, we observed that MDA levels of BAT decreased in the females raising larger litters compared with those supporting 1 pup. But this does not conflict the observation that UCP_1_ and UCP_3_ lower ROS production. The plausible explanation is that BAT activity and capacity are suppressed during lactation to protect the mother against hyperthermia [2,59,61–63]. The decreased activity of BAT was characterized by the notable lower rate of metabolism in mitochondria, consequently resulted in the lack of elevation of ROS levels. These findings in the striped hamster may suggest that lactation has a different effect on a variety of tissues in the context of the cellular metabolic rate. This may be a potential factor resulting in the tissue-dependent changes of oxidative stress in response to reproduction.

## In Summary

The results from the present study may suggest that the cost of reproduction has negligible effect on oxidative stress or even attenuated oxidative stress in some active tissues, including BAT, liver and skeletal muscle, in striped hamsters. However, the cost of reproduction might cause a significant increase of oxidative stress in the brain, indicating that oxidative stress in response to reproduction is tissue dependent. RMR significantly increased, but BAT UCP_1_ mRNA expression decreased with the experimentally increasing litter size. This suggests that there is no positive relationship between metabolic rate and ROS levels. The decreased activity of BAT is characterized by the notable lower rate of metabolism in mitochondria and down-regulated mRNA expression, consequently resulting in the lack of elevation of ROS levels. Lactation has different effects on a variety of tissues in the context of the cellular metabolic rate, which may be a potential factor resulting in the tissue-dependent changes of oxidative stress in response to reproduction.
